# Recent Advances in Tannic Acid (Gallotannin) Anticancer Activities and Drug Delivery Systems for Efficacy Improvement; A Comprehensive Review

**DOI:** 10.3390/molecules26051486

**Published:** 2021-03-09

**Authors:** Rana A. Youness, Rabab Kamel, Nermeen A. Elkasabgy, Ping Shao, Mohamed A. Farag

**Affiliations:** 1 The Molecular Genetics Research Team, Department of Pharmaceutical Biology, Faculty of Pharmacy andBiotechnology, German University in Cairo, Cairo 12622, Egypt; rana.youness21@gmail.com; 2 Pharmaceutical Technology Department, National Research Centre, Cairo 12622, Egypt; drrababk@hotmail.com; 3 Department of Pharmaceutics and Industrial Pharmacy, Faculty of Pharmacy, Cairo University, Kasr El-Aini Street, Cairo 11562, Egypt; nermeen.ahmed.elkasabgy@pharma.cu.edu.eg; 4 Department of Food Science and Technology, Zhejiang University of Technology, Hangzhou 310014, China; pingshao325@zjut.edu.cn; 5 Pharmacognosy Department, College of Pharmacy, Cairo University, Kasr El Aini St., Cairo 11562, Egypt; 6 Chemistry Department, School of Sciences & Engineering, The American University in Cairo, New Cairo 11835, Egypt

**Keywords:** tannic acid, anticancer, combinatorial effects, drug delivery systems, nanoparticles, molecular mechanisms

## Abstract

Tannic acid is a chief gallo-tannin belonging to the hydrolysable tannins extracted from gall nuts and other plant sources. A myriad of pharmaceutical and biological applications in the medical field has been well recognized to tannic acid. Among these effects, potential anticancer activities against several solid malignancies such as liver, breast, lung, pancreatic, colorectal and ovarian cancers have been reported. Tannic acid was found to play a maestro-role in tuning several oncological signaling pathways including JAK/STAT, RAS/RAF/mTOR, TGF-β1/TGF-β1R axis, VEGF/VEGFR and CXCL12/CXCR4 axes. The combinational beneficial effects of tannic acid with other conventional chemotherapeutic drugs have been clearly demonstrated in literature such as a synergistic anticancer effect and enhancement of the chemo-sensitivity in several resistant cases. Yet, clinical applications of tannic acid have been limited owing to its poor lipid solubility, low bioavailability, off-taste, and short half-life. To overcome such obstacles, novel drug delivery systems have been employed to deliver tannic acid with the aim of improving its applications and/or efficacy against cancer cells. Among these drug delivery systems are several types of organic and metallic nanoparticles. In this review, the authors focus on the molecular mechanisms of tannic acid in tuning several neoplastic diseases as well as novel drug delivery systems that can be used for its clinical applications with an attempt to provide a systemic reference to promote the development of tannic acid as a cheap drug and/or drug delivery system in cancer management.

## 1. Introduction

The process of carcinogenesis consists of complex steps that end up by transforming a normal functional cell into an unresponsive aggressive neoplastic one [1]. This process involves a series of transition states starting with initiation, promotion, progression and to end up with metastasis to other distant organs [2]. Alterations at the genetic and the epigenetic levels are the main fuels that promote such transitions in cancer development [3].

Yet, the introduction of nutraceuticals or phytochemicals in the field of oncology has shown promising results concerning prevention, treatment and/or harnessing of that disease [4,5,6]. Moreover, nutraceuticals’ potential has exceeded the limits, and very recent reports have highlighted their potential in tuning the immunogenic profile of cancer cells to be radically eliminated by the immune cells in a process called immune surveillance [6,7]. A positive correlation between a healthy diet that is rich in fruits and vegetables, and the prevention of health-daunting diseases such as cancer, has been well evidenced, and is partially ascribed to polyphenols as dietary bioactive class [8]. Natural compounds were reported to halt carcinogenesis through several molecular mechanisms such as the regulation of cell cycle, cell apoptosis, migration, invasion, cancer stemness phenotype and other molecular signaling pathways [4].

Tannins are phenolic compounds resulting from the secondary metabolism in several plants of high economic and ecological values. Tannic acid (TA) is a hydrolysable tannin present in several natural sources such as grapes, green tea, coffee, and others [9]. Concerning the physiochemical properties of TA, its molecular weight is 1701.2 g/mol with weak acidic properties and a strong astringent taste. TA exhibits a myriad of medicinal benefits such as anticancer, antioxidant, anti-inflammatory and neuro-protective effects [10]. In contrast, TA can interact with biopolymers and macromolecules by cross-linking due to its hydroxy and carboxy groups [11], posing it as a promising pharmaceutical candidate. This review focuses on TA potential as an anticancer agent and its possible incorporation in several drug delivery systems to improve its delivery, efficacy and clinical application.

TA has been recently casted as one of the promising polyphenolic phytochemicals that has a well-defined role in each transition step in the process of carcinogenesis [12]. It retains several pharmacological actions that pose it as a potential antitumorigenic agent mediated via various mechanisms of actions including radical scavenging [13], anti-oxidant [14] and anti-inflammatory effects [15]. However, little is known about the molecular signaling pathways tuned by such phytochemical compound in different malignant scenarios.

**Figure 1:**
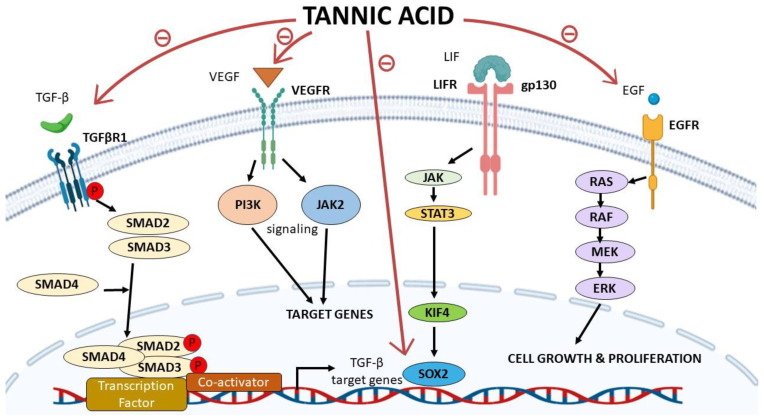
Tannic acid (TA) acts as a repressor for multiple proteins essential in several oncological signaling pathways. TA inhibits SMAD-dependent gene transcription in response to TGF-β, therefore inhibiting the transcription of TGF-β target genes. TA also inhibits VEGF/VEGFR, inhibiting a major angiogenesis signaling pathway in cancer. Moreover, TA inhibits the expression of SOX2 gene and inhibits EGF/EGFR signaling pathway and consequently cell growth and proliferation.

**Figure 2:**
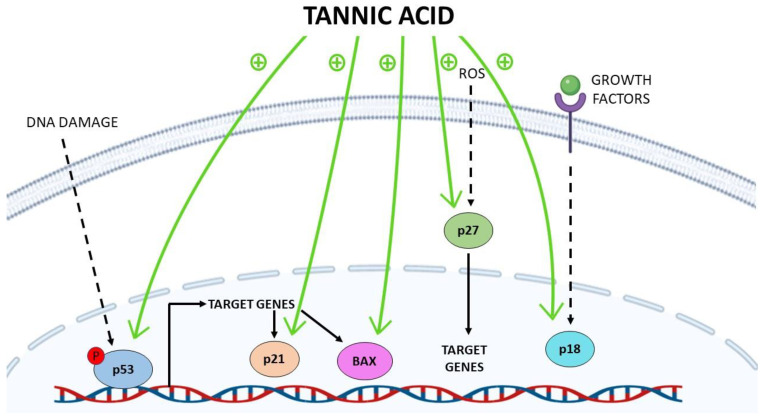
Induction of tumor suppressor proteins by tannic acid (TA). TA enhances the phosphorylation of p53, thereby increasing the expression of its target genes such as p21 and BAX. Moreover, on another level, TA could stimulate both p21 and BAX gene expression directly. TA also stimulates the gene expression of p27 and p18.

Recently, a growing volume of evidence has been piled in literature supporting the versatile ability of TA in harnessing the oncological process. Novel molecular mechanisms have been drawn downstream TA, thus confirming its potential anticancer action, posing TA as an adjuvant therapy in combination with other conventional chemotherapeutic agents. In this review, our main aim is to unveil the molecular mechanisms underlying the anticancer properties of TA and its intracellular target proteins in different cancer types, as summarized in Table 1, and Figure 1 and Figure 2.

## 2. TA in Lung Cancer

Globally, lung cancer is the leading cause of cancer-related deaths [30]. Statistically, around 80% of lung cancer cases fall under the non-small-cell lung carcinoma (NSCLC) category. TA was found to repress several hallmarks of lung cancer *in vitro* such as the repression of cellular viability, invasion, colony forming ability, migration and cancer cells’ stemness [16,21]. This was verified in two NSCLC cell lines known as A549 and H1299, and with no significant toxicity effects on human bronchial epithelial cells (BEAS-2B). Mechanistically, this was achieved through the direct binding of TA to transforming growth factor-β1 (TGF-β1), leading to a simultaneous repression of TGF-β1 and TGF-β1 receptor (TGF-β1R), turning off all signaling pathways downstream TGF-β1/TGF-β1R, as shown in Figure 1 [16]. Moreover, a repression of several epithelial-to-mesenchymal transition (EMT) mediators such as N-cadherin, type-1-collagen, fibronectin, and vimentin in A549 cell lines was also observed. Additionally, a significant deactivation of Smad2/3/Akt/ERK1/2/JNK1/2 and p38 mediators was detected [16]. Likewise, a repression of VEGF/VEGFR2-related pathway was confirmed [18]. It should be noted that TA effects on NSCLC cells have been extended to affect vital cell cycle regulatory proteins such as Cyclin D1, p53, p21, p18, BAX, BCL-2 [18]. In such context, another recent study performed by Nipin *et al.* also confirmed the pro-apoptotic effects of TA in NSCLC cells via inducing cell cycle arrest at G0/G1 phase [21], where cell cycle arrest at this particular phase has been a repeatedly reported phenomena exhibited by TA in lung cancer cells [16,18]. Also, the same group has reported that TA could reduce the stemness of NSCLC cells through reducing the sphere formation ability of A549 cells concurrent with a reduction of SOX2, OCT4 and NANOG expression that represent vital markers for cancer stem cells (see Figure 1). Likewise, a marked reduction in the percentage of cells over-expressing CD133 was evidenced [21]. Collectively, these experimental evidences support the anticancer activity of TA against lung cancer and specifically NSCLC cells. However, it should be noted that all reported studies were based on *in vitro* testing of TA against NSCLC cells; thus, this urges more research on TA molecular mechanisms to confirm such results using *in vivo* models. It is essential in parallel to perform selective toxicity studies for TA to confirm the selective anticancer effects of TA against lung cancer and to promote its use in clinical trials in the future.

## 3. TA in Breast Cancer

Breast cancer (BC) is considered the most common cause of cancer-related mortalities among women [30]. BC is a heterogeneous disease that involves various molecular subtypes such as hormone receptors-positive tumors, human epidermal growth factor (HER-2) over-expressed tumors and the most aggressive subtype, triple-negative tumors [31,32]. TA has been described as an anticancer agent in all the previously mentioned molecular subtypes, found to induce apoptosis in hormone receptors-positive BC cells (MCF-7) [33], HER-2 positive BC cells (BT474) [34] and the triple-negative BC cells (MDA-MB-231) [22] mainly by activating a series of caspases such as caspase 3/7 and caspase 9, as shown in Figure 2 and inhibiting the overly expressed fatty acid synthase in BC cells [22]. Mechanistically, TA was found to negatively regulate the oncogenic JAK/STAT signaling pathway in BC cells regardless of its molecular subtype, as indicated in Figure 1 [23]. Furthermore, it was also found to deactivate the epidermal growth factor receptor (EGFR) and thus to affect the canonical and non-canonical STAT pathways resulting in G1 arrest and activation of intrinsic apoptotic pathways in BC cells [23]. In a more comprehensive study, TA was reported to alleviate TGF-β induced EMT and NF-κB activation, thus inhibiting cancer stem cells’ (CSCs) activity in murine mouse model, highlighting TA as a promising therapeutic approach for BC patients [17]. It is also worth mentioning that TA was found not to inhibit the normal human epithelial cells (MCF10) growth posing TA as a selective anticancer agent in BC [33]. TA was identified as a novel selective CXCL12/CXCR4 antagonist being able to selectively inhibit CXCL12-induced migration (IC_50_, 7.5 μg/mL) in MDA-MB-231 cells, as shown in Figure 1 and Table 1 [24]. Nonetheless, TA was found to ameliorate doxorubicin (potent anti-cancer agent)-induced cardiotoxicity and to further potentiate its anticancer activity against MDA-MB-231 cells [35]. Formulation based on TA to ameliorate doxorubicin side effects shall be discussed later in this review. In a more translational approach, TA was tested in a combinatorial approach with paclitaxel, a standard chemotherapeutic agent for BC as well as other solid malignancies such as ovarian, pancreatic and NSCLC. A crystal-clear synergistic effect was observed upon co-treatment of MDA-MB-231 cells with TA and paclitaxel on the proliferation, colony-forming ability, migration and invasion capacities when compared to the solo-treatment of MDA-MB-231 cells with Paclitaxel [36]. Therefore, such intensive investigational studies of TA against different BC cell lines, mouse models and in combinational treatment protocols highly suggest the pan-tumor suppressor activity of TA against multiple subtypes of BC, including the most aggressive BC subtype (triple-negative BC).

## 4. TA in Colorectal Cancer

Colorectal cancer (CRC) is the third most common cancer worldwide after lung and breast cancers among both sexes, yet is the second most common cause of cancer-related mortalities in both males and females collectively [30]. CRC was one of the earliest types of cancers that was reported to respond to the anticancer activity of TA. TA was found to reduce the cellular viability of CaCo-2 cell lines in a dose- and time-dependent manner through increasing the apoptotic index of such cells as well as inducing the Bak and Fas-associated protein with death domain (FADD) protein percentage ratios. Such evidence unveiled a potential role of TA in inducing apoptosis in CRC through mitochondrial and death receptor pathways [29]. In a more comprehensive study, TA was found to act as a selective inhibitor of pyruvate kinase isoenzyme M2 (PKM2) in CRC cells resulting in an attenuation of the CRC cellular proliferation capacity, as shown in Figure 1 [25]. In a more clinical approach, TA was reported to alleviate several side effects exhibited by Oxaliplatin anticancer agent, where it was found that a combinatory approach of dual delivering TA and Oxaliplatin showed several advantages over conventional usage of Oxaliplatin treatment alone. TA/Oxaliplatin combination resulted in a synergistic reduction in the tumor size of the induced human CRC *in-vivo*, improved the quality of life and prolonged the survival time of mice. This collectively supports the anticancer activity of TA CRC both *in vitro* and *in vivo*, yet a more detailed description of the molecular activity of TA in CRC cells is highly needed to exactly recognize the array of downstream signals which might propagate inside CRC cells.

## 5. TA in Liver Cancer

Liver cancer is the third leading cause of cancer-related deaths among males [30]. TA was found to act in a synergistic manner with Cisplastin (a potent antitumor agent) in preventing liver cancer progression *in vitro* through inducing the mitochondrial-mediated apoptosis [37]. Moreover, Mhlanga *et al.* have recently reported the effect of TA on HepG2 cells where they found out that TA induced apoptotic pathways, increased reactive oxygen and nitrogen species while decreased antioxidants expression. Consequently, DNA fragmentation via caspase-dependent and -independent pathways has occurred followed by instant cell death, as shown in Figure 2 [28]. It is also worth mentioning that TA has been described as a hepato-protective and anti-fibrotic agent where it was found to decrease ALT and AST serum levels *in vivo* and to act as fibrinolytic agent through altering the TIMP/MMP balance and thus inhibit the activation of the hepatic stellate cells (chief fibrinogenic cell in liver) [26]. Collectively, TA can be described as dually acting anti-fibrotic and anticancer agent in liver cancer, suggesting its high potential in alleviating steatotic and liver cancer patients.

## 6. TA in Ovarian Cancer

Epithelial ovarian cancer is regarded as the most common and fatal ovarian cancer of gynecological malignancies [27]. Advanced ovarian cancer patients are the least fortunate in terms of effective therapeutic approaches, where the backbone treatment is cytoreductive surgery and Cisplatin-based chemotherapy. Yet, a large proportion of patients exhibit an innate or acquired type of resistance against Cisplastin-based treatment protocols [38]. In such context, TA was investigated in combination with Cisplastin in different human ovarian carcinoma cell lines such as Cisplatin-resistant (SKOV-3 CDDP/R) and Cisplatin-sensitive (SKOV-3). Such combinational approach was found to induce apoptosis and increase DNA damage in both ovarian carcinoma cell lines [27]. Such effect was not exhibited in normal human ovarian cell, suggesting for its safety. Mechanistically, it was found that TA enhances the activity of Cisplatin in ovarian carcinoma cells through the inhibition of poly(ADP-ribose) glycohydrolase (PARG) expression, increasing the accumulation of poly(ADP-ribose) (pADPr), following the release of apoptosis-inducing factors, and the activation of caspase-3, as shown in Figure 1 and Figure 2, thus drawing a clear mechanism for how/why TA is recommended in a combinatorial approach with Cisplatin in the treatment of advanced ovarian carcinoma, especially in the case of Cisplatin-resistant patients [27].

## 7. TA in Pancreatic Cancer

Pancreatic cancer (PanCa) is one of the most lethal carcinomas, with a 5 year survival rate of only 7% [39]. PanCa is typically diagnosed at a late stage of the disease, rendering the surgical option and leaving PanCa patients as victims for the side effects associated with the ineffective chemotherapeutic agents such as 5-Flurouracil, Gemcitabine, and Irinotecan [40], highlighting an urgent need to foster other drug regimens or combinations to improve patients’ survival rates. The implication of TA in a combinational strategy with the 3 FDA-approved chemotherapeutic agents—5-Flurouracil, Gemcitabine, and Irinotecan—in a nano-technological fashion was examined. TA was used as part of an innovative approach of highly stable nano-complexes specifically targeting pancreatic cancer adenocarcinoma (PDAC) cell lines (HPAF-II and PANC-1) resulting in a marked repression of PDAC cellular proliferation and clonogenicity [40].

## 8. TA in Prostate Cancer

Prostate cancer (PCa) is the second most common cause of cancer-related mortalities after lung cancer in males [30]. TA was found to retract cellular growth, clonogenic, invasive, and migratory capacities of PCa cells [19,41]. Mechanistically, TA was found to induce ER activation stress response (Protein kinase R-like endoplasmic reticulum kinase (PERK) and inositol requiring enzyme 1 (IRE1)), and alter their downstream regulatory proteins (ATF4, Bip, and PDI) expression in PCa cell lines, Table 1 [19]. Nonetheless, TA treatment was reported to induce the expression of the apoptosis-associated markers, i.e., Bak, Bim, cleaved caspase 3, and cleaved PARP (Figure 2). On the other hand, a marked repression of the pro-survival proteins (Bcl-2 and Bcl-xL) was also reported in the same experiment, as shown in Figure 1. It is also important to note that TA had an effect on cell cycle in PCa cells where an arrest of G1/S phase was reported together with an elevation of the tumor suppressor proteins p18 and p21 concurrent with suppression of cyclin D1 expression. Also, a marked attenuation of MMP2 and MMP9 was reported to suggest for the antimetastatic potential of TA against PCa cell lines [19]. Nevertheless, such effect has yet to be tested using animal models to be more conclusive. In a more comprehensive study, a dose-dependent attenuation of C4-2, DU145, and PC-3 cellular proliferation was reported in response to TA administration. Additionally, an induction of ROS species has been evidenced in PCa cells as a result of induction of endoplasmic reticulum stress signaling pathway upon TA treatment [42]. Nonetheless, TA was also found to alter lipid metabolism and disrupt the cellular and the nuclear membranes in PCa cells [42]. Collectively, this highly suggests TA as a potential antitumorigenic weapon in PCa.

## 9. TA in Gingival Cancer

Gingival squamous cell carcinoma (GSCC) is a rare type of cancer which comprises < 10% of all head and neck squamous cell carcinomas [20]. GSCC has a high probability of metastasis and specially to the bones [43]. TA was reported to harness GSCC cellular proliferation *in vitro.* To unravel the molecular mechanisms underlying such anticancer activity against YD-38 cells, a deactivation of STAT3 transcriptional activity was coupled to TA treatment [20]. Moreover, TA treatment resulted in an induction of P53 transcript levels, resulting in G1 arrest in YD-38 cells. Molecularly, it was reported that TA treatment resulted in a marked attenuation of the positive regulators of cell cycle such as cyclin D1, cyclin E and CDK-4, and at the same time resulted in a transcriptional activation of the negative regulators of the cell cycle such as p21 and p27, as shown in Figure 2 [20], thus presenting a potential anticarcinogenic effect against GSCC cells mainly through altering vital cell cycle regulators.

## 10. TA-Based Pharmaceutical Formulations

**Figure 3:**
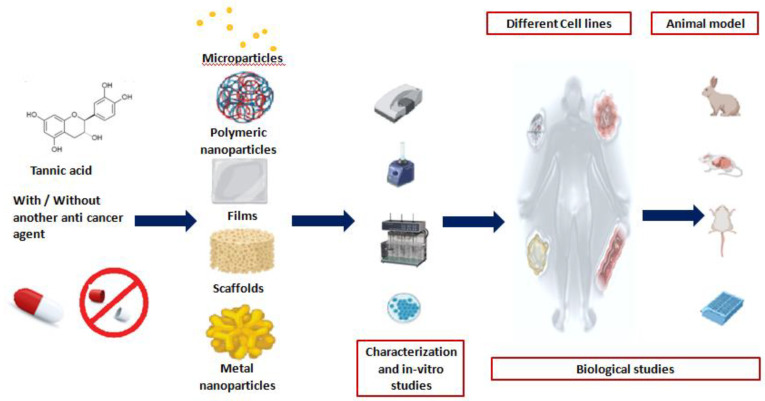
Steps of pharmaceutical investigation including preparation, characterization and evaluation of versatile TA-based pharmaceutical formulations using *in vitro*- and *in vivo*-based assays.

Our second focus in this review was to provide a roadmap for the clinical application of TA. TA is a promising naturally occurring substance which can be designated as a dual function ingredient in pharmaceuticals, being an effective anticancer agent as well as a pharmaceutical excipient having the potential to act as a cross-linker aiding in the preparation of versatile formulations, as illustrated in Figure 3.

Of the several formulations of TA targeting its anticancer action, poly(TA) microparticles were prepared via cross-linking using trimethyl-ol-propane-triglycidyl ether and glycerol diglycidyl ether and to exhibit an antioxidant, antimicrobial and cytotoxic effects. The prepared particles were effective against A549 cancerous cells comparable to that of the anticancer drug Cisplatin (36% and 34% cellular viability, respectively) while the cellular viability was 66% in the case of linear TA at a dose of 37.5 μg mL^−1^ [44].

Likewise, TA was cross-linked with different biological macromolecules to prepare matrices tested for their potential anticancer effects. TA solution was incorporated by diffusion into collagen type I beads stabilized via hydrogen bonding between the amines of collagen peptide backbone and phenolic group of TA. This prepared device was able to act as a biocompatible cell scaffold for the regeneration and reconstruction of breast tissue with prolonged anticancer activity. In addition, the small diameter of the beads (~1 mm) allows its subdermal injection offering a non-invasive reconstructive option for outpatients [45]. In the same context, TA was used as a cross-linking agent to prepare collagen-based breast tissue scaffolds. The concentration of TA solution used influenced apoptosis in MCF-7 BC cells as well as toxic effect on D1 mesenchymal stem-like stromal healthy cells, suggestive that TA level cross-linking can be changed to provide an optimum effect [46].

Thin films composed of different ratios of chitosan and TA mixture (80/20, 50/50 and 20/80) were tested *in vitro* on different cell lines such as MNT-1, SK-MEL-28, Saos-2, HaCaT and BMSC. The results showed that the strongest influence was recorded for MNT-1 cells versus weakest for BMSC cell line. It was observed that films with higher surface roughness (Chitosan: TA 80/20) had the highest ability to inhibit cell growth [47]. Likewise, TA cross-linked collagen gels inhibited the proliferation of melanoma cells and with gel stiffness found halting of tumor proliferation and progression occurred [48].

## 11. Combinational Approach of TA with Conventional Chemotherapeutic Agents in Pharmaceutical Formulations

Combined with other anticancer agents, TA deployment in nanotechnology has been introduced in several studies for cancer therapeutics. Below are displayed some examples of these combinations, as summarized in Table 2 and explained below.

TA is considered as a polydentate ligand that could bind to various metal ions to form stable metal complexes. Such metal nanoparticles have several advantages including anticancer properties. For instance, gold nanoparticles (AuNPs) have shown several applications in cancer treatment [54] as well as incorporation in drug delivery systems [55]. TA-stabilized gold nanoparticles (TA/AuNP) were synthesized, and showed higher cytotoxic activity against different cancer cell lines (HCT116, MCF7 and HepG2) as compared to free TA. Such potentiation is likely due to the generation of more efficient ROS by the TA/AuNPs in HCT116 cells compared to TA leading to a decrease in IC_50_ value. Additional advantage of TA/AuNPs lies in its improved stability up to 50 days and less toxicity on normal cells (HEK 293) compared to TA, with an IC_50_ values at 80.45 and 16.67μM, respectively. However, Nag *et al*.’s study revealed that TA has high binding affinity towardsAkt oncogenic protein, which repressed the expression of Akt, and hence inhibited the survival of colon cancer cells as explained earlier in this review [56]. In another study, Fe^+3^–TA nanoparticles were prepared using a low-cost, reproducible iron-mediated self-assembly process revealing that the higher uptake of Fe^+3^–TA nanoparticles by HepG2 cells can result in autophagic cell death. Consequently, prepared nanoparticles offer a promising approach for imaging and further the treatment of liver cancer [57].

The combination of Oxaliplatin, an anti-colorectal cancer agent with TA for the preparation of polymeric nanoparticles using poly-lactic acid-10R5-PLA (PLAR) using the solvent evaporation technique [49]. For achieving more targeted and sustained action with effective therapeutics (drug & TA) dose at the site of action, the formed nanoparticles were loaded in thermosensitive hydrogels with delayed degradability at the site of action. Invitro studies on CT26 cells verified the synergistic inhibitory action of TA and Oxaliplatin with combination index < 1.Colorectal peritoneal carcinomatosis mouse invivo models were further used to assess the anticancer efficacy of the developed thermosensitive hydrogels administered intra-peritoneal at a dose of 10 mg/kg for Oxaliplatin and TA individually. The prepared nanoparticles loaded-hydrogels extended the median survival life up to 38 days compared to free drug (27 days) and hydrogel loaded with blank nanoparticles (21 days) [49]. The synergistic effect of TA with chemotherapeutics was revealed in another study for the treatment of breast cancer via the preparation of Paclitaxel/TA nanoparticles using solvent evaporation. The TA-based nanoparticles possessed small particle size of ca.102 nm, high encapsulation efficiency values > 95%, good short-term stability (6 days) at ambient temperature and excellent binding efficiency of TA with Paclitaxel. These bindings were due to hydrogen bonding and electrostatic attractions as verified using infrared spectroscopy [36]. Assembled TA nanoparticles showed superior cellular uptake of the drug in MDA-MB-231 cells compared to the plain drug at 95% versus 57%, respectively, which might be attributed to TA ability to partially inhibit the ATPase and in turn P-glycoprotein [58]. Furthermore, nanoparticles possessed higher anti-proliferative effect compared to plain drug post 2 days of treatment on MDA-MB-231 and MCF7 breast cancer cells with significantly lower IC_50_ values by 41% and 13% for the aforementioned cell lines, respectively. Although these findings are promising, long-term stability studies should be conducted for these fabricated nanoparticles for application at an industrial scale in pharmaceutical companies.

The cross-linking ability of TA was exploited with modified pectin to fabricate nanocomplexes through hydrogen bonding, offering a promising platform for pancreatic cancer treatment [40]. Pectin can target cancer cells via its γ-galactose units [59], whereas TA can act as a bridge capturing hydrophilic (gemcitabine) and hydrophobic (5-Flurouracil & Irinotecan) anticancer drugs through self-assembly.Drug-loaded nanocomplexes possessed superior anticancer properties compared to free drugs against HPAF-II and PANC-1 cells, indicating their efficacy to actively deliver the drugs to cancer cells as determined by lower IC_50_ values and increased cellular uptake as detected by using flow cytometry [40]. Similar significant growth-inhibiting effect against A549 and H1299 cells —verified by 2- to 2.5-fold decrease in IC_50_ values—was obtained with drug (Gemcitabine, Carboplatin Oririnotecan)-loaded nanocomplexes constructed from the interaction between TA and mice lung fluid through hydrogen bonding attributed to the adherence of lung fluid on the surface of TA highlighting the efficient drug carrier properties of TA [50].

With regards to the ability of TA to modulate the physicochemical properties of drug delivery systems, as well as to decrease cytotoxic drug side effects, one study reported the combination of TA with the widely used anticancer agent doxorubicin hydrochloride [60]. Doxorubicin suffers from an initial burst release due to its high water solubility [61], necessitating the use of higher drug doses that could lead to cardiotoxicity [62]. TA was used to limit its toxicity through the construction of freeze-dried Doxorubicin-loaded polymeric nanoparticles prepared by the co-assembly between Doxorubicin, TA (hydrogen donating molecule) [63], and neutral poly (2-methyl-2-oxaozline). The nanoparticles possessed good stability in physiological environment—screened by negligible change in particle size—after storage in phosphate buffer saline (PBS) (pH 7.4) for 6 days. Additionally, the nanoparticles showed sustained drug release profile at different release rates at different pH values (5 & 7.4), with the higher release rate observed in acidic medium [51], which is considered an added value as it mimics the relative acidic pH values inside tumor cells [64,65].The inhibition capacity of the nanoparticles on HeLa cell lines was nearly similar to the free drug at a concentration of 5 mg/mL drug [51], confirming the success of such co-assembly. However, invivo studies on experimental animals should be further conducted to elucidate its therapeutic applicability and monitoring its side effects. Although the enhanced drug release in acidic medium is favored for cancer therapy, sometimes because of the physicochemical properties of drugs as well as the route of administration, this concept can be modified as previously mentioned with Paclitaxel-loaded TA/polyvinylpyrilidone nanoparticles (54 nm) prepared using flash nano-precipitation. The nanoparticles showed the formation of microaggregates (2 µm) at acidic conditions (pH 2) due to the low ionization of TA and hence formation of strong hydrogen bonding. In contrast, quick dissociation and release of drug was observed at higher pH values of 6.8 or 7.4 [66]. Such pH sensitivity could protect acid-sensitive drugs like Paclitaxel [67] from degradation inside the stomach and achieve site-specific drug release in the intestine to enhance its oral bioavailability. This effect was further confirmed in MCF-7 tumor-bearing mice, where more enhanced tumor inhibition rate at 86% compared to 78% was observed after oral administration of nanoparticles given at a dose of 20 mg/Kg drug compared to Taxol^®^ injection (positive control; 10 mg/Kg), respectively [66]. Such enhanced inhibition effect might also be attributed to the ability of TA to inhibit P-glycoprotein as previously reported [58]. TA/polyvinylpyrilidone/zoledronic acid self-assembled nanoparticles particle size of ca. 400 nm, targeted mice bones and prostate tumor tissues in an exvivostudy as well as enhanced drug delivery to tumor cells [68].

Another study reported the combination of TA with hydrophobic anticancer drugs, i.e., nobelitin as nanoparticles assembled by adsorbing TA on zein/nobelitin aggregates. TA was further cross-linked with metal ions (Fe^3+^ & Al^3+^) to form a cross-linked coat with good long-term stability as denoted by no significant changes in particle size between fresh and stored ones. Besides, this cross-linked coat enhanced the entrapment efficiency of the drug by nearly 50% compared to zein nanoparticles lacking the presence of such coat with a complete sustained drug release at pH 7.4 within 25 h compared to free drug (within 5 h). Excellent anticancer response against H1299 cell line was recorded for coated nanoparticles at a nobelitin concentration of 80 µg/mL with good cytocompatibility [52]. This co-assembly between TA/metal ions using zein as a polymer could offer an effective carrier for other hydrophobic drugs for cancer treatment in the future. Another study reported the cross-linking of TA with FeCl_3_·6H_2_O to be used as a coat for Paclitaxel nanoparticles. Paclitaxel nanocomplexes showed sustained drug release behavior which were pH-dependent, i.e., the release efficiency of Paclitaxel was reached 51% & 30% at pH 5.2 & 7.4, respectively, within one week. Additionally, the nanocomplexes demonstrated excellent anticancer effect against A549 cells with reduced IC_50_ values compared to crude drug and uncoated Paclitaxel nanoparticles, likely attributed to the improved stability of Paclitaxel nanocomplexes in neutral pH [53] as well as improved endocytosis of the complexes [69]. Collectively, these results highlight the multifaceted role of TA and sheds light onto the diversity of essential oils that contain considerable amount of TA to be considered as anticancer agent beside its well-known anti-microbial activity [70,71,72].

Considering the merits of nanotechnology, more research should be directed towards new developments to improve cancer drug therapy based on nanotechnology. Nanotransformers with quick response to different biological environments inside the body have been recently introduced through tailoring their physical properties [73]. Nano-transformers prepared by co-assembling TA (hydroxyl group donor for interactions), Doxorubicin and Indocyanine green (photothermal agent) showed enhanced proton prompted hydrophobic-hydrophilic transformation associated with size conversion characters. In brief, the preparation method relied on the assembly of TA with doxorubicin through π−π interactions and electronic interactions, and then Indocyanine green was assembled with drug/TA assembly through π−π interactions and electrostatic interactions in water as a safe green solvent [73].

The transformation hypothesis is based mainly on the conversion of the prolonged blood-circulating hydrophilic nanoassembly (bypass phagocytosis by reticuloendothelial system) to the hydrophobic micron-sized particles inside the acidic pH of the tumor cell. These micron-sized particles were able to rupture the lysozyme [74,75] then re-transform to the hydrophilic nanoform in the cytoplasm where they could release their drug payloads. The optimum nanotransformers possessed small particle size (nearly 74 nm) and with an encapsulation efficiency of 23% and 35% for Doxorubicin and Indocyanine, respectively. The nanotransformersenhanced significantly the decrease in tumor weights in MCF7 xeno-grafted tumor mice group compared to doxorubicin, indocyanine green administered group, which might be attributed to the nanotransformers acting to enhance the synergistic effect of Doxorubicin and Indocyanine green [70]. Despite the success offered by these nanotransformers, further studies altering weight proportions of the components are required to enhance the encapsulation efficiency of the drug to reach higher levels. Besides, invitro and invivo studies on experimental animal models should be further conducted to explore the anticancer role of TA-based nanotransformers compared to others prepared using another –OH group donating materials i.e., L-ascorbic acid, D-mannitol and hydroxylpropyl-β-cyclodextrin.

## 12. Conclusions

**Figure 4:**
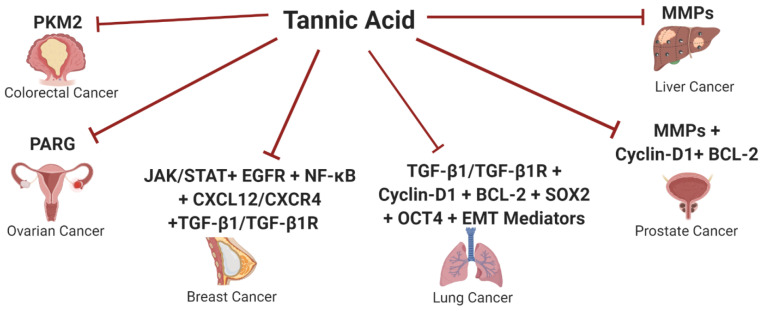
Schematic Representation of Tannic acid potential anticancer activity against several solid malignancies. This figure represents a summary of the potential role of Tannic acid (TA) as an anticancer agent through repressing several oncogenic signaling pathways and tumor-promoting factors in different solid malignancies such as colorectal, ovarian, breast, lung, prostate and liver cancers.

This review represents a comprehensive overview on the deployment of TA in cancer therapy. The authors tried to shed light onto the versatile ability of TA to halt the malignant transformation process in several dominating solid malignancies such as lung, breast, liver, pancreatic, prostate, ovarian and gingival carcinomas, as summarized in Figure 4. TA appears to act as a multifunctional player turning off several oncogenic signaling pathways simultaneously such as VEGF, TGF-β1 pathways together with the repression of vital oncogenic mediators such as MMPs and several EMT mediators, Figure 4. Nonetheless, TA has also been proven to act as a potent cell cycle regulator in different malignancies. Such promising antimalignant actions pose TA as a promising, cheap and safe anticancer agent to act on different molecular targets. TA showed selective promising anticancer activities against two of the most dominating cancers worldwide, which are lung and breast cancers. In a more translational approach, this review focused on the combinational strategies of TA with other conventional chemotherapeutic agents, which showed promising results in different studies, including some nanotechnology-based formulations. Nanotechnology approaches with enhanced delivery capability for therapeutics have shown myriad benefits in cancer treatment. However, several bio-barriers, i.e., poor tumor penetration, inactivation of drugs by lysozymes, can mitigate the efficacy of therapeutics transport, hence developing innovative and different nanotechnology approaches is still needed to reap the full merits of nanotechnology applications, i.e., nanocomplexes and nanotransformers.

Furthermore, this review also paves the road for the clinical application of such potentially abundant cheap naturally occurring polyphenolic compound through highlighting its potential incorporation into the pharmaceutical industries. Asides from its promising anticancer activity, the chemical structure of TA (hydroxyl donating molecule) presents the potential to be used as a strong cross-linking agent, hence acting as a dual-functional pharmaceutical ingredient.

Several studies have reported the preparation of various successful anticancer TA-based drug delivery systems; however, more experimental and clinical studies should be conducted in the future to prove its efficacy. Additionally, this promising compound should be well exploited in designing different TA-based phytotherapeutics based on novel technologies aiming to tackle its low bioavailability and short half-life in the human body as well as to create safe, effective and low-cost remedies for cancer treatment. Nonetheless, detailed analytical studies should be performed to screen for herb–drug interactions with tannic acid as its polyphenolic nature could have several interferences with other drugs. Also it is worth mentioning that there are no studies that addressed the preparation of TA-based personalized medications using the 3D printing technique. This emerging technology could offer a good opportunity for the preparation of natural inks for the development of cheap 3D prints as a part of the future digital pharmacies.

## Figures and Tables

**Table 1:** Molecular targets of tannic acid (TA) in various neoplastic diseases.

Effect	Molecular Target	Cancer Type	References
Inhibition	TGF-β1/TGF-β1R axis	Lung Cancer and Breast Cancer	[16,17]
	EMT Mediators	Lung Cancer	[16]
	VEGF/VEGFR axis	Lung Cancer	[18]
	Cyclin D1	Lung Cancer, Prostate Cancer, Gingival Cancer	[18,19,20]
	BCL-2	Lung Cancer, Prostate Cancer	[18,19]
	SOX2	Lung Cancer	[21]
	OCT4	Lung Cancer	[21]
	NANOG	Lung Cancer	[21]
	Fatty acid synthase	Breast Cancer	[22]
	JAK/STAT signaling pathway	Gingival Cancer, Breast Cancer,	[20,23]
	epidermal growth factor receptor (EGFR)	Breast Cancer	[23]
	NF-Κb	Breast Cancer	[17]
	CXCL12/CXCR4	Breast Cancer	[24]
	pyruvate kinase isoenzyme M2 (PKM2)	Colorectal Cancer	[25]
	MMPs	Prostate Cancer, Liver Cancer,	[19,26]
	poly(ADP-ribose) glycohydrolase (PARG)	Ovarian Cancer	[27]
Induction	p53	Lung Cancer, Gingival Cancer	[18,20]
	p21	Lung Cancer, Prostate Cancer, Gingival Cancer	[18,19,20]
	P27	Gingival Cancer	[20]
	p18	Lung Cancer, Prostate Cancer	[18,19]
	BAX	Lung Cancer, Prostate Cancer	[18,19]
	Caspases	Prostate Cancer, Breast Cancer, Liver Cancer, Ovarian Cancer	[19,22,28], [27]
	Bak and FADD ratio	Colorectal Cancer	[29]
	ER stress response (Protein kinase R-like endoplasmic reticulum kinase (PERK) and inositol requiring enzyme 1 (IRE1)	Prostate Cancer	[19]

**Table 2:** Examples of several combinational Approaches of TA and other anticancer agents.

Drug	Dosage Form	Cell Line	Most Important Key Finding	References
Oxaliplatin	Polymeric nanoparticle	CT26	Synergistic inhibitory effect of oxaliplatin and TA	[49]
Paclitaxel	Nanoparticle	MDA-MB-231	Reduced IC_50_ & increased cellular uptake of the drug compared to free drug	[36]
Gemcitabine, 5-Flurouracil &irinotecan	Nanocomplex	HPAF-II and PANC-1	Lower IC_50_ value& increased cellular uptake for the drugs	[40]
Gemcitabine, carboplatin &irinotecan	Nanocomplex	A549 and H1299	Enhanced decrease in IC_50_ from drug-loaded nanocomplex fabricated from TA and mice lung fluid	[50]
Doxorubicin hydrochloride	Polymeric nanoparticle	HeLa	Similar inhibitory action of the nanoparticles compared to the free drug at the same concentration of 5 mg/mL	[51]
Nobelitin	Coated nanoparticle	H1299	Improved anticancer effect as well as good cytocompatibility at nobelitin concentration of 80 µg/ mL	[52]
Paclitaxel	Nanocomplex	A549	Lower IC_50_ values compared to both the pure drug and uncoated nanoparticles	[53]

## Data Availability

Not applicable.

## Sample Availability

Samples of the compounds are not available from the authors.
